# CT-Derived Muscle and Adipose Tissue Characteristics and Survival in Advanced NSCLC Treated with First-Line Immunotherapy-Based Regimens

**DOI:** 10.3390/cancers18172857

**Published:** 2026-09-03

**Authors:** Blerina Resuli, Amanda Tufman, Friederike Völter, Diego Kauffmann-Guerrero, Paula Mras, Paola Arnold, Clemens Bleistein, Jürgen Behr, Victoriya Vasileva, Lalith Kumar Shiyam Sundar, Jens Ricke, Clemens Cyran, Wolfgang G. Kunz, Matthias P. Fabritius, Nabeel Mansour

**Affiliations:** 1 Department of Medicine V, Thoracic Oncology Centre Munich, LMU University Hospital, LMU Munich, 80539 Munich, Germany; amanda.tufman@med.uni-muenchen.de (A.T.); diego.kauffmannguerrero@med.uni-muenchen.de (D.K.-G.); paula.mras@med.uni-muenchen.de (P.M.); paola.arnold@med.uni-muenchen.de (P.A.); clemens.bleistein@med.uni-muenchen.de (C.B.); juergen.behr@med.uni-muenchen.de (J.B.); 2 Comprehensive Pneumology Center Munich (CPC-M), German Center for Lung Research (DZL), Bavarian Center for Cancer Research (BZKF), 81675 Munich, Germany; friederike.voelter@med.uni-muenchen.de; 3 Department of Nuclear Medicine, LMU University Hospital, LMU Munich, 80539 Munich, Germany; 4 Department of Internal Medicine IV, LMU University Hospital, LMU Munich, 80539 Munich, Germany; 5 Department of Radiology, LMU University Hospital, LMU Medizin, Ludwig-Maximilians Universität München, 80539 Munich, Germany; victoriya.vasileva@med.uni-muenchen.de (V.V.);; 6 Digital Transformation in Radiology (DIGIT-X) Lab, Department of Radiology, Ludwig Maximilian University of Munich, 80539 Munich, Germany

**Keywords:** NSCLC, sarcopenia, CT-derived skeletal muscle, body composition, artificial intelligence

## Abstract

Background: Host-related factors such as skeletal muscle and adipose tissue characteristics are increasingly recognized as determinants of outcome in advanced non-small cell lung cancer (aNSCLC). However, the prognostic relevance of integrated body composition phenotypes in patients receiving first-line immunotherapy-based treatment remains unclear. Methods: We retrospectively analyzed patients with aNSCLC treated with first-line immune checkpoint inhibitors (ICI) alone or combined with chemotherapy (ICI–CT) between October 2018 and December 2023 at LMU University Hospital. AI-derived CT biomarkers included skeletal muscle volume index (SMVI), skeletal muscle density (SMD), visceral adipose volume index (VAVI), and subcutaneous adipose volume index (SAVI), normalized for height. Individual body composition parameters were analyzed as standardized continuous variables. For exploratory phenotype analyses, SMVI and VAVI were categorized using cohort-specific sex-stratified median values. An integrated body composition phenotype was defined as “high-muscle/low-visceral-adiposity” (high SMVI/low VAVI) or “low-muscle/high-visceral-adiposity” (low SMVI/high VAVI), with patients not meeting either definition classified as having an intermediate phenotype. Multivariable Cox regression adjusted for age, sex, treatment modality, and metastatic burden evaluated associations with progression-free survival (PFS) and overall survival (OS). Results: Of 116 screened patients, 103 with evaluable baseline CT imaging were included. The cohort predominantly comprised ever-smokers (81.6%) and patients with adenocarcinoma (62.1%). Higher SMD (per one standard deviation increase) was associated with improved overall survival (HR 0.72, 95% CI 0.52–1.00; *p* = 0.047), and higher VAVI was associated with a higher risk of death (HR 1.29, 95% CI 1.00–1.65; *p* = 0.048). Neither SMD nor VAVI was significantly associated with PFS, and SMVI and SAVI were not significantly associated with OS or PFS. In the exploratory integrated phenotype analysis, median OS was 29.5 months in the high-muscle/low-visceral-adiposity group, 16.2 months in the intermediate group, and 19.3 months in the low-muscle/high-visceral-adiposity group; however, the overall Kaplan–Meier comparison was not statistically significant (log-rank *p* = 0.385). Conclusions: AI-derived CT body composition assessment may provide complementary prognostic information in patients with aNSCLC receiving first-line immunotherapy-based treatment. Higher skeletal muscle density was associated with a lower risk of death, whereas higher visceral adiposity was associated with a higher risk of death. The exploratory integrated phenotype analysis did not demonstrate a consistent risk gradient across phenotype categories. These findings require validation in larger prospective cohorts before clinical implementation.

## Simple Summary

Patients with advanced non-small cell lung cancer who receive immunotherapy-based treatment often experience markedly different outcomes, highlighting the need for additional prognostic biomarkers. Routine computed tomography (CT) scans contain valuable information beyond tumor assessment, including measurements of skeletal muscle and body fat. In this retrospective study, we used an artificial intelligence-based tool to automatically quantify body composition from routinely acquired CT images. We investigated both individual body composition parameters and integrated phenotypes combining skeletal muscle volume and visceral adiposity. Higher skeletal muscle density, a marker of better muscle quality, was associated with improved overall survival. Higher visceral adiposity was associated with poorer overall survival. In the exploratory integrated phenotype analysis using cohort-specific, sex-stratified median values, patients with a high-muscle/low-visceral-adiposity phenotype showed numerically favorable survival, although a consistent survival gradient across phenotype groups was not observed. These findings suggest that automated analysis of routine CT scans may provide complementary prognostic information without requiring additional examinations or increasing patient burden. Because this was a single-center retrospective study with a relatively small cohort, our results should be considered exploratory and require confirmation in larger, prospective studies before routine clinical implementation.

## 1. Introduction

Immune checkpoint inhibitors (ICIs) targeting the programmed death protein 1/programmed death-ligand 1 (PD-1/PD-L1) pathway have significantly improved survival outcomes in patients with advanced non-small cell lung cancer (aNSCLC) and currently represent the standard first-line treatment for most patients without actionable oncogenic alterations [1,2,3]. Despite these advances, only a subset of patients derives durable clinical benefit from ICIs, highlighting the need for reliable biomarkers to improve patient selection and prognostic stratification.

PD-L1 tumor expression remains the most widely used biomarker for treatment selection; however, its predictive performance is suboptimal. A substantial proportion of patients with high PD-L1 expression fail to respond to immunotherapy, whereas meaningful responses may also occur in tumors with low or absent PD-L1 expression [4]. Similarly, the clinical utility of tumor mutational burden (TMB) has been inconsistent across studies and remains limited in routine practice [5]. These limitations have stimulated interest in host-related biomarkers, including systemic inflammation, nutritional status, and body composition, which may complement tumor-based biomarkers and are readily assessable in routine clinical practice [6,7].

Advances in artificial intelligence (AI)-based image segmentation now enable reproducible and automated quantification of skeletal muscle and adipose tissue compartments from routinely acquired CT examinations, facilitating standardized body composition analysis [8,9]. Growing evidence suggests that CT-derived body composition parameters are associated with treatment tolerance, physical function, and survival across several malignancies, including advanced NSCLC [10,11,12].

Among body composition biomarkers, low skeletal muscle mass, commonly assessed by the skeletal muscle index (SMI), has been associated with poorer clinical outcomes. However, muscle quantity alone may not fully reflect cancer-related metabolic impairment. Skeletal muscle density (SMD), a surrogate marker of myosteatosis, and visceral adiposity have also been linked to chronic inflammation, immune dysregulation, and unfavorable oncologic outcomes [13,14,15,16,17,18]. These body composition features may reflect the systemic metabolic and inflammatory milieu, which is increasingly recognized as an important determinant of antitumor immune responses and treatment efficacy. Consequently, CT-derived body composition biomarkers have emerged as promising candidates for prognostic stratification in patients receiving ICIs. However, existing evidence remains heterogeneous, with most studies evaluating individual body composition parameters rather than their combined prognostic value. Moreover, data derived from AI-based body composition analysis in patients with aNSCLC receiving contemporary first-line immunotherapy-based regimens is still limited.

Therefore, we investigated whether AI-derived CT measures of skeletal muscle quantity, muscle quality, and adipose tissue distribution were associated with survival in patients with advanced NSCLC receiving contemporary first-line ICI-based therapy. In addition, we explored whether an integrated phenotype combining skeletal muscle volume and visceral adiposity could provide complementary prognostic information beyond individual body composition parameters. Given the absence of validated volumetric thresholds for these AI-derived measures, the integrated phenotype analysis was considered exploratory.

## 2. Methods

### 2.1. Study Design and Patients

This retrospective observational study included consecutive patients with aNSCLC treated with first-line ICI-based therapy at LMU Hospital Munich, Germany, between October 2018 and December 2023. The retrospective consecutive-cohort design was selected to evaluate the prognostic relevance of CT-derived body composition measures under real-world treatment conditions and to minimize selection based on clinical outcome. No patient selection was performed according to treatment response or survival outcome.

Eligible patients had histologically or cytologically confirmed stage IIIC–IV NSCLC according to the AJCC 8th edition TNM classification and received first-line ICI monotherapy or ICI combined with platinum-based chemotherapy in routine clinical practice. Patients harboring actionable oncogenic driver alterations, including EGFR mutations and ALK or ROS1 rearrangements, were excluded.

Availability of a baseline PET/CT examination performed before treatment initiation was required for inclusion. The CT component of the PET/CT scan was used for automated body composition analysis. Patients without evaluable baseline imaging or with inadequate image quality for reliable segmentation were excluded.

Clinical and pathological data were retrieved from electronic medical records and included age, sex, smoking status, histology, treatment regimen, and survival outcomes. Follow-up information was obtained from institutional clinical records and cancer registries.

The study was conducted in accordance with the Declaration of Helsinki and approved by the Ethics Committee of Ludwig Maximilian University of Munich (reference number 476-16 UE; 5 August 2016). The requirement for written informed consent was waived owing to the retrospective design and use of anonymized data.

### 2.2. CT-Based Body Composition Analysis

Baseline CT scans acquired as part of routine clinical staging prior to initiation of systemic therapy were used for body composition analysis. All examinations were derived from pretreatment PET/CT studies, and the CT component was used for quantitative analysis. For each examination, the reconstruction series with the lowest available slice thickness using a soft-tissue kernel was selected to optimize spatial resolution.

CT-derived body composition analysis was performed using a fully automated artificial intelligence (AI)-based segmentation workflow integrated into the Visage Imaging platform (Visage Imaging GmbH, Berlin, Germany). Tissue segmentation was conducted using the Multi-Organ Objective Segmentation (MOOSE) pipeline, a deep learning–based semantic segmentation framework built on the nnU-Net architecture, enabling automated three-dimensional delineation of anatomical structures from CT datasets [9].

The software automatically identified the third lumbar vertebral level (L3), a validated anatomical landmark for body composition assessment, and performed volumetric segmentation of skeletal muscle, visceral adipose tissue (VAT), and subcutaneous adipose tissue (SAT). Volumetric measurements were generated across the entire L3 slice thickness, allowing assessment of tissue volumes rather than conventional single-slice areas.

The automated segmentation pipeline generated quantitative measurements of skeletal muscle volume, visceral adipose tissue volume, subcutaneous adipose tissue volume, and skeletal muscle density (SMD). Skeletal muscle volume index (SMVI), visceral adipose volume index (VAVI), and subcutaneous adipose volume index (SAVI) were calculated by normalizing the corresponding tissue volumes to patient height (cm^3^/m^2^).

**Figure 1:**
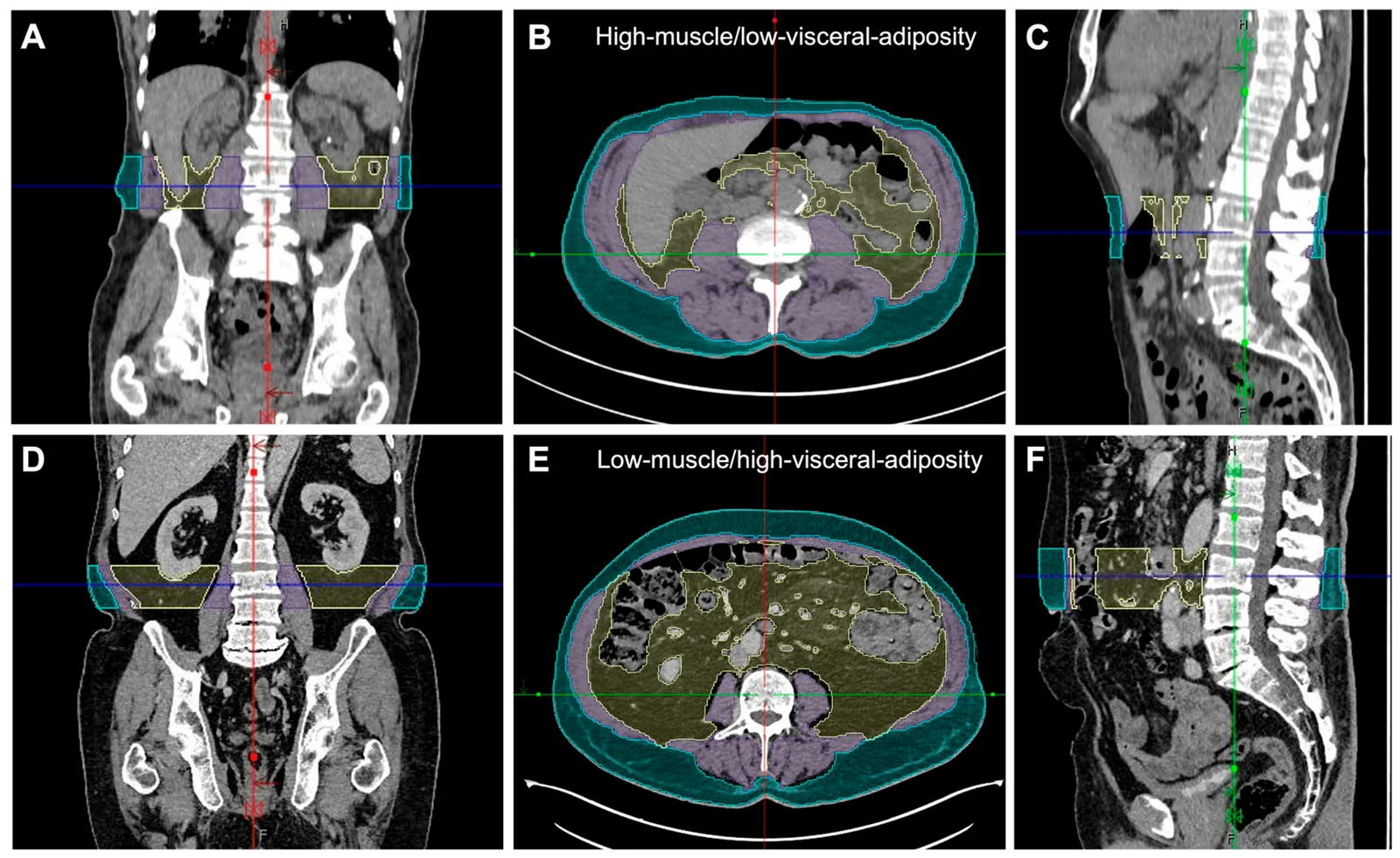
Representative AI-derived CT body composition phenotypes in patients with advanced NSCLC receiving first-line immunotherapy-based treatment. Panels (**A**–**C**) demonstrate a high-muscle/low-visceral-adiposity phenotype characterized by preserved skeletal muscle volume and relatively low visceral adiposity (high SMVI/low VAVI; SMVI 282.6 cm^3^/m^2^, VAVI 132.8 cm^3^/m^2^). Panels (**D**–**F**) illustrate the low-muscle/high-visceral-adiposity phenotype (low SMVI/high VAVI; SMVI 210.9 cm^3^/m^2^, VAVI 431.6 cm^3^/m^2^). Coronal, axial, and sagittal CT reconstructions are shown from left to right. Automated deep learning–based segmentation at the level of the third lumbar vertebra (L3) identified skeletal muscle (purple), visceral adipose tissue (yellow), and subcutaneous adipose tissue (turquoise) compartments from routine pretreatment CT imaging.

All body composition analyses were performed on baseline imaging acquired prior to initiation of immunotherapy-based systemic treatment to ensure assessment of pretreatment physiological status. Automated segmentations underwent visual quality control by an experienced radiologist (N.M.) to confirm anatomical plausibility and correct landmark identification. Segmentations considered anatomically implausible or affected by incorrect L3 localization were reviewed for potential exclusion/correction. Representative AI-derived CT body composition segmentations of the high-muscle/low-visceral-adiposity and low-muscle/high-visceral-adiposity phenotypes are shown in Figure 1.

### 2.3. Clinical Endpoints and Statistical Analysis

The primary objective of this study was to evaluate the prognostic significance of CT-derived body composition biomarkers, including SMD, SMVI, VAVI, and SAVI, in patients with aNSCLC treated with first-line ICI-based regimens. SMVI was used as a measure of skeletal muscle quantity, whereas SMD was considered a marker of skeletal muscle quality.

The primary endpoints were overall survival (OS) and progression-free survival (PFS). OS was defined as the time from initiation of first-line systemic therapy to death from any cause, with patients alive at data cutoff censored at the date of last documented follow-up. PFS was defined as the time from treatment initiation to radiologically documented disease progression or death from any cause, whichever occurred first. Patients without progression or death were censored at the date of last documented follow-up.

Individual body composition parameters (SMD, SMVI, VAVI, and SAVI) were analyzed primarily as standardized continuous variables (per one standard deviation increase). For the exploratory integrated phenotype analysis, SMVI and VAVI were additionally categorized using cohort-specific sex-stratified median values. The median SMVI was 256.98 cm^3^/m^2^ in men and 205.43 cm^3^/m^2^ in women, while the median VAVI was 268.21 cm^3^/m^2^ in men and 169.90 cm^3^/m^2^ in women. Within each sex, values at or above the corresponding median were classified as high and values below the median as low. Because validated volumetric cutoffs for these AI-derived body composition measures are unavailable, this sex-stratified median-based categorization was used solely for exploratory phenotype classification. An integrated body composition phenotype was defined using SMVI and VAVI categories. Patients with high SMVI and low VAVI were classified as having a high-muscle/low-visceral-adiposity phenotype, whereas patients with low SMVI and high VAVI were classified as having a low-muscle/high-visceral-adiposity phenotype. Patients with all other SMVI/VAVI combinations were categorized as having an intermediate phenotype. Because measures of muscle strength and physical performance were unavailable, low SMVI was not considered equivalent to a clinical diagnosis of sarcopenia.

Continuous variables are presented as means with standard deviations or medians with interquartile ranges (IQRs), as appropriate, and categorical variables as frequencies and percentages. Baseline characteristics were additionally summarized according to integrated body composition phenotypes. Group comparisons were performed using the Kruskal–Wallis’s test for continuous variables and the chi-square test or Fisher’s exact test, as appropriate, for categorical variables.

Associations between body composition parameters and survival outcomes were evaluated using Cox proportional hazards regression models, with hazard ratios (HRs) and corresponding 95% confidence intervals (CIs). Multivariable models were prespecified to adjust for age, sex, treatment modality (ICI monotherapy versus chemoimmunotherapy), and metastatic burden. To minimize model overfitting, each body composition parameter and the integrated phenotype were entered separately into the same core clinical model.

Survival distributions were estimated using the Kaplan–Meier method and compared using the log-rank test. The principal analyses included 103 patients with evaluable baseline CT-derived body composition measurements. Missing covariate data were handled using complete-case analysis for multivariable models, and the number of patients and events included in each model was reported. Patients without an OS or PFS event remained in the analyses and were censored at the date of last documented follow-up.

All statistical tests were two-sided, and *p*-values < 0.05 were considered statistically significant. Because multiple exploratory body composition and sensitivity analyses were performed, no formal adjustment for multiple testing was applied. Statistical analyses were performed using R software (version 4.4.2; R Foundation for Statistical Computing, Vienna, Austria).

## 3. Results

### 3.1. Patient Characteristics and Treatment Distribution

Between October 2018 and December 2023, 116 consecutive patients with aNSCLC who received first-line ICI-based therapy at our institution were screened for eligibility. Thirteen patients were excluded because baseline CT imaging was unavailable or unsuitable for reliable automated body composition analysis owing to insufficient image quality. The final study cohort comprised 103 patients with evaluable baseline CT imaging and complete AI-derived body composition measurements (Figure 1).

Baseline demographic and clinicopathological characteristics are summarized in Table 1, including stratification according to the integrated body composition phenotype. The mean age at treatment initiation was 65.0 years (SD 11.7 years), and most patients were male (65/103, 63.1%). A history of tobacco exposure was common, with 42 patients (40.8%) identified as current smokers and 42 patients (40.8%) as former smokers. Ten patients (9.7%) were never-smokers, while smoking status was unavailable in nine patients (8.7%).

Regarding tumor histology, adenocarcinoma represented the predominant subtype and was diagnosed in 64 patients (62.1%), whereas squamous-cell carcinoma accounted for 27 cases (26.2%). The remaining 12 patients (11.7%) had other NSCLC histologies. PD-L1 tumor proportion score (TPS) was broadly distributed across clinically relevant categories, with PD-L1 expression < 1% observed in 32 patients (32.7%), TPS 1–49% in 31 patients (31.6%), and TPS ≥ 50% in 35 patients (35.7%). With respect to first-line treatment, 22 patients (21.4%) received ICI monotherapy, while 81 patients (78.6%) were treated with combined chemoimmunotherapy.

### 3.2. Clinical Outcomes According to Treatment Strategy

The association between treatment modality and survival outcomes was evaluated because treatment modality was included as a prespecified adjustment variable in subsequent multivariable analyses.

For overall survival, Kaplan–Meier analysis demonstrated no significant difference between patients treated with ICI monotherapy and those receiving chemoimmunotherapy (log-rank *p* = 0.57). Consistent with these findings, univariable Cox proportional hazards regression analysis showed that treatment modality was not associated with OS (HR 1.18, 95% CI 0.67–2.08; *p* = 0.57).

For progression-free survival, Kaplan–Meier analysis revealed a non-significant trend toward shorter PFS among patients treated with chemoimmunotherapy compared with those receiving ICI monotherapy (log-rank *p* = 0.09). In univariable Cox regression analysis, chemoimmunotherapy was associated with a non-significant increase in the risk of disease progression or death (HR 1.61, 95% CI 0.93–2.81; *p* = 0.09). However, treatment modality was not independently associated with progression-free survival after multivariable adjustment.

Overall, no statistically significant association was observed between treatment modality and either OS or PFS. Accordingly, treatment modality was retained as a prespecified adjustment variable in subsequent multivariable analyses.

### 3.3. CT-Derived Body Composition Markers and Integrated Body Composition Phenotypes

Automated AI-based body composition analysis was successfully completed in all 103 evaluable baseline CT datasets obtained from pretreatment PET/CT examinations. Quantitative assessment revealed substantial heterogeneity in body composition across the study population. The median SMVI was 241.7 cm^3^/m^2^ (IQR, 207.5–280.1), median VAVI was 226.6 cm^3^/m^2^ (IQR, 135.0–364.4), and median SAVI was 300.2 cm^3^/m^2^ (IQR, 208.8–402.5). Skeletal muscle density, reflecting muscle quality and intramuscular fat infiltration, also demonstrated considerable variability among patients, consistent with the heterogeneous metabolic profiles observed in advanced NSCLC.

To investigate the combined prognostic value of skeletal muscle quantity and visceral adiposity, patients were classified into exploratory integrated body composition phenotypes based on sex-specific median SMVI and VAVI values. Patients with high SMVI and low VAVI were classified as having a high-muscle/low-visceral-adiposity phenotype, whereas patients with low SMVI and high VAVI were classified as having a low-muscle/high-visceral-adiposity phenotype. Patients not fulfilling either definition were assigned to an intermediate phenotype.

**Figure 2:**
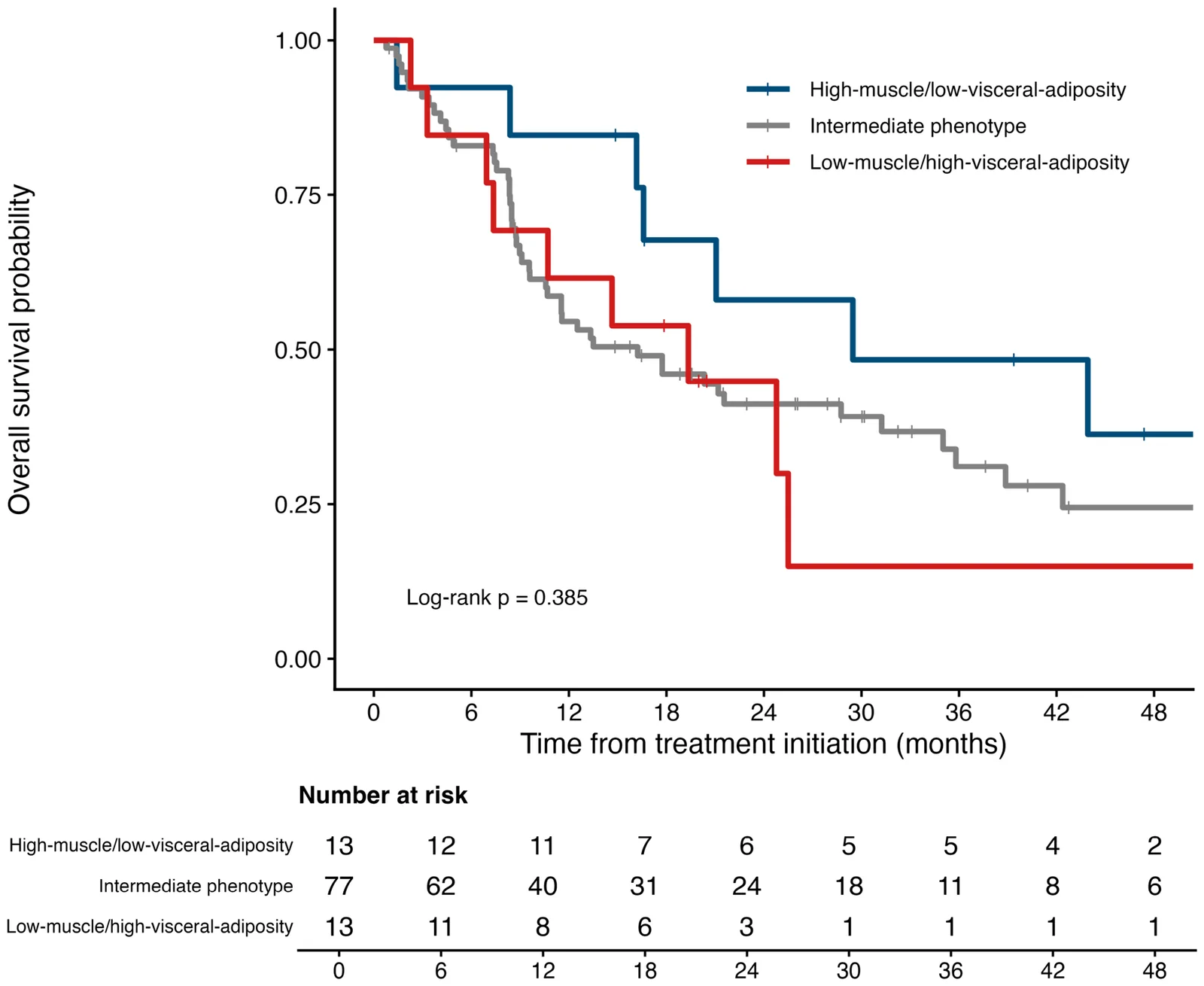
Overall survival according to integrated body composition phenotypes. Kaplan–Meier curves showing overall survival in patients with aNSCLC treated with first-line ICI-based therapy according to high-muscle/low-visceral-adiposity, intermediate, and low-muscle/high-visceral-adiposity phenotypes.

**Figure 3:**
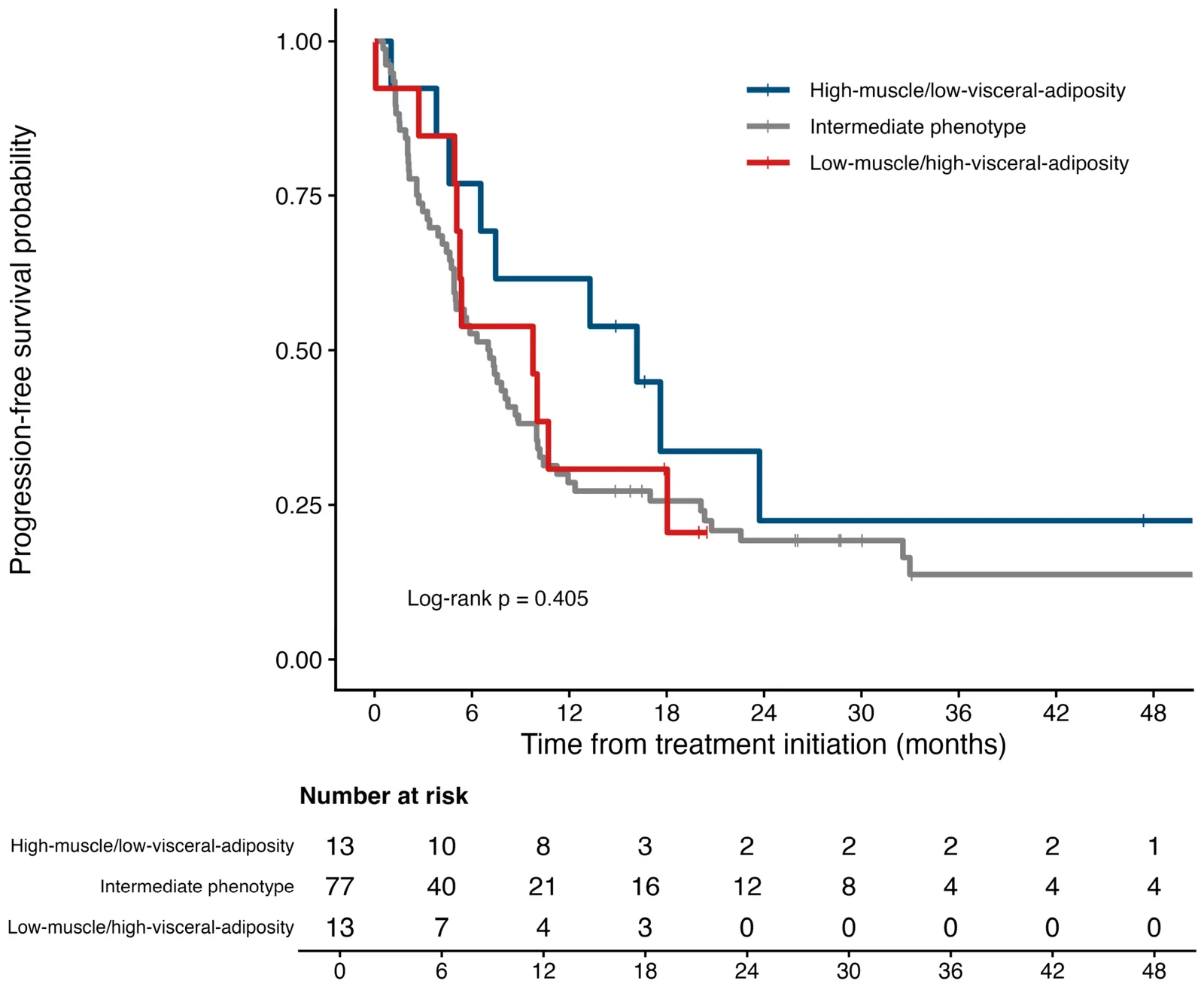
Progression-free survival according to integrated body composition phenotypes. Kaplan–Meier curves for progression-free survival according to integrated body composition phenotypes.

Median OS was 29.5 months in patients with a high-muscle/low-visceral-adiposity phenotype, 16.2 months in patients with an intermediate phenotype, and 19.3 months in patients with a low-muscle/high-visceral-adiposity phenotype. Although median OS differed numerically across integrated body composition phenotype groups defined using sex-specific cutoffs, the overall unadjusted Kaplan–Meier comparison was not statistically significant (log-rank *p* = 0.385). In the multivariable Cox model, patients with an intermediate phenotype had a higher mortality risk compared with the high-muscle/low-visceral-adiposity reference group (HR 2.50, 95% CI 1.04–6.02; *p* = 0.041), whereas the low-muscle/high-visceral-adiposity phenotype was not significantly associated with mortality (HR 2.15, 95% CI 0.76–6.13; *p* = 0.151). These findings did not demonstrate a consistent stepwise risk gradient across the three phenotype categories (Figure 2). Given the small number of patients in the extreme phenotype groups, these findings should be interpreted as exploratory. Kaplan–Meier analysis of progression-free survival according to the integrated body composition phenotypes defined using sex-specific cutoffs did not demonstrate statistically significant differences between groups (log-rank *p* = 0.405; Figure 3).

**Figure 4:**
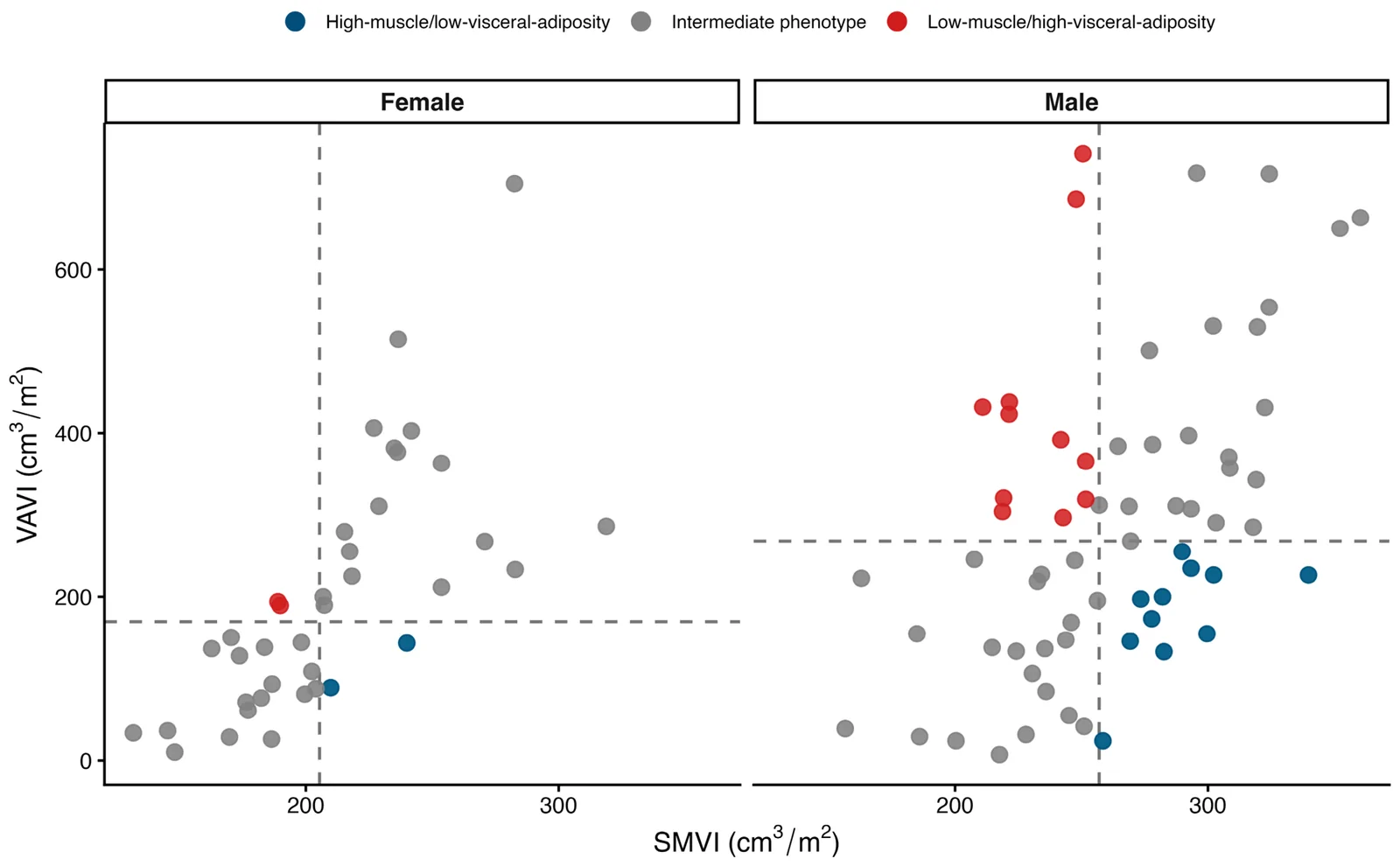
Distribution of integrated body composition phenotypes according to skeletal muscle volume index (SMVI) and visceral adipose volume index (VAVI), stratified by sex. Dashed vertical and horizontal lines indicate the cohort-specific sex-stratified median values for SMVI and VAVI, respectively. Patients were classified as high-muscle/low-visceral-adiposity, low-muscle/high-visceral-adiposity, or intermediate phenotype according to their position relative to the corresponding sex-specific median values.

The distribution of integrated body composition phenotypes according to SMVI and VAVI values, stratified by sex, is illustrated in Figure 4. Considerable variability in both skeletal muscle volume and visceral adiposity was observed throughout the cohort. Patients with a high-muscle/low-visceral-adiposity phenotype clustered predominantly within the high-SMVI and low-VAVI ranges, whereas patients with a low-muscle/high-visceral-adiposity phenotype were concentrated within the low-SMVI and high-VAVI regions of the distribution. Intermediate phenotypes occupied the remaining areas, highlighting the broad spectrum of body composition profiles among patients receiving first-line immunotherapy-based treatment.

**Figure 5:**
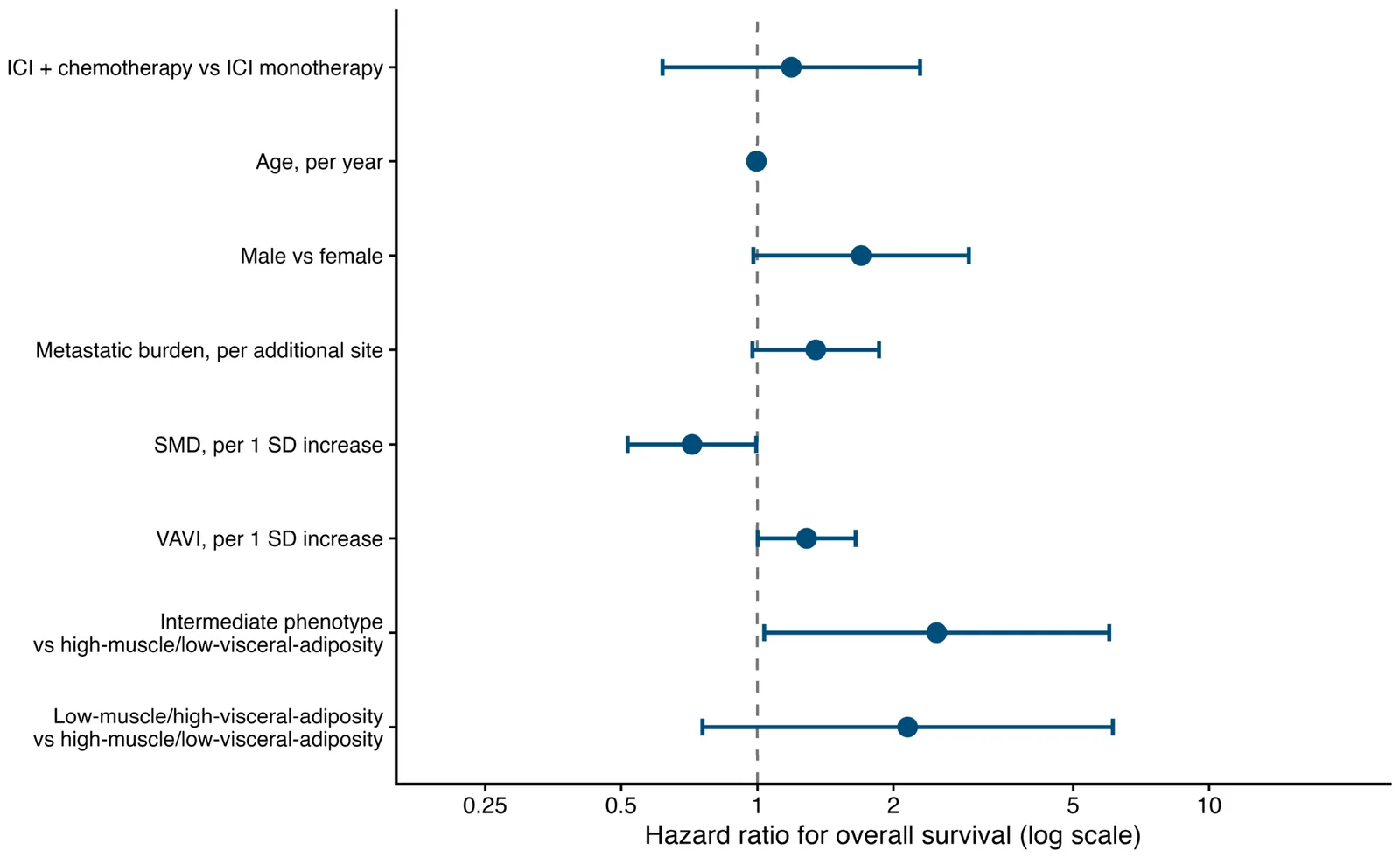
Adjusted hazard ratios for overall survival. Forest plot summarizing the associations of clinical variables and selected body composition parameters with overall survival. Clinical variables were derived from the baseline multivariable model, whereas SMD, VAVI, and the integrated body composition phenotype were evaluated in separate multivariable models adjusted for age, sex, treatment modality, and metastatic burden. Hazard ratios are presented with 95% confidence intervals.

To determine whether these phenotypes provided prognostic information beyond established clinical factors, multivariable Cox regression analyses adjusted for age, sex, treatment modality, and metastatic burden were performed. Compared with the high-muscle/low-visceral-adiposity reference group, patients with an intermediate phenotype had a higher risk of death (HR 2.50, 95% CI 1.04–6.02; *p* = 0.041), whereas the low-muscle/high-visceral-adiposity phenotype was not significantly associated with mortality (HR 2.15, 95% CI 0.76–6.13; *p* = 0.151). For progression-free survival, the intermediate phenotype was associated with a higher risk of progression or death compared with the high-muscle/low-visceral-adiposity reference group (HR 2.18, 95% CI 1.01–4.70; *p* = 0.048), whereas the low-muscle/high-visceral-adiposity phenotype was not significantly associated with PFS (HR 1.87, 95% CI 0.72–4.85; *p* = 0.196). Given the absence of a consistent stepwise risk gradient across the three phenotype categories, these phenotype-based findings should be interpreted as exploratory. Complete-case multivariable analyses included 100 patients, with 64 OS events and 81 PFS events. Among the individual CT-derived body composition parameters, SMD and VAVI were significantly associated with overall survival in the primary multivariable analyses. After adjustment for age, sex, treatment modality, and metastatic burden, each one-standard-deviation increase in SMD was associated with a lower risk of death (HR 0.72, 95% CI 0.52–1.00; *p* = 0.047), whereas each one-standard-deviation increase in VAVI was associated with a higher risk of death (HR 1.29, 95% CI 1.00–1.65; *p* = 0.048). In contrast, neither SMVI (HR 0.93, 95% CI 0.69–1.27; *p* = 0.660) nor SAVI (HR 1.02, 95% CI 0.77–1.36; *p* = 0.870) was significantly associated with overall survival. None of the four continuous body composition parameters was significantly associated with progression-free survival: SMD (HR 0.86, 95% CI 0.65–1.14; *p* = 0.301), SMVI (HR 0.88, 95% CI 0.67–1.14; *p* = 0.331), VAVI (HR 1.22, 95% CI 0.96–1.54; *p* = 0.100), and SAVI (HR 0.96, 95% CI 0.75–1.24; *p* = 0.775). The results of the primary multivariable analyses are summarized in Figure 5. Given the established association between low muscle attenuation and adverse clinical outcomes, additional exploratory analyses were performed using previously reported sex-specific thresholds for myosteatosis (<33 HU for men and <28 HU for women). Using these criteria, myosteatosis was identified in 61% of male patients and 43% of female patients. However, dichotomous classification according to these predefined cutoffs was not significantly associated with overall survival (HR 1.25, 95% CI 0.77–2.04; *p* = 0.36) or exploratory progression-free survival (HR 1.00, 95% CI 0.63–1.59; *p* = 0.99). Importantly, body-composition thresholds may be population-dependent, and cut-offs derived or validated in a specific cohort may not be directly generalizable to populations with different demographic and clinical characteristics, including sex distribution, ethnicity, age, BMI, and cancer type. This heterogeneity should be considered when comparing results across studies and highlights the need for validation in independent populations.

Sex was not independently associated with overall survival after multivariable adjustment (HR 1.70, 95% CI 0.98–2.94; *p* = 0.059).

Overall, these findings indicate that among the individual CT-derived body composition parameters, higher skeletal muscle density was associated with a lower risk of death, whereas higher visceral adipose volume index was associated with a higher risk of death. The exploratory integrated phenotype analysis using sex-specific cutoffs showed numerically favorable survival in the high-muscle/low-visceral-adiposity group; however, no consistent stepwise risk gradient across the three phenotype categories was observed. These findings support further investigation of muscle quality and visceral adiposity as complementary components of body composition in patients receiving first-line immunotherapy-based treatment.

## 4. Discussion

In this real-world cohort of patients with aNSCLC treated with first-line ICI-based regimens, CT-derived body composition profiling identified substantial heterogeneity in survival outcomes. Among the individual body composition parameters, higher SMD was associated with a lower risk of death in the primary multivariable analysis, whereas higher VAVI was associated with a higher risk of death. In contrast, SMVI and SAVI were not significantly associated with overall survival. The exploratory integrated phenotype analysis showed numerically favorable survival in patients with a high-muscle/low-visceral-adiposity phenotype; however, the overall Kaplan–Meier comparison was not statistically significant, and no consistent stepwise risk gradient was observed across the three phenotype categories. Together, these findings suggest that muscle quality and visceral adiposity may provide complementary information for characterizing host-related body composition profiles associated with survival in patients receiving first-line ICI-based therapy.

The exploratory analysis of integrated body composition phenotypes provided additional insight into the combined distribution of skeletal muscle volume and visceral adiposity in this cohort. Patients with a high-muscle/low-visceral-adiposity phenotype showed numerically favorable survival; however, the overall Kaplan–Meier comparison was not statistically significant, and the low-muscle/high-visceral-adiposity phenotype was not significantly associated with mortality compared with the high-muscle/low-visceral-adiposity reference group after multivariable adjustment. Notably, SMVI alone was not independently associated with overall survival, whereas higher VAVI was associated with a higher risk of death in the primary multivariable analysis. Accordingly, the absence of a consistent stepwise risk gradient across the phenotype categories argues against interpreting the combined phenotype as an established prognostic classification. These findings support the concept that considering skeletal muscle and visceral adiposity together may provide a more comprehensive characterization of body composition than evaluating isolated radiologic variables alone [6,19,20,21], although the prognostic relevance of the integrated phenotype requires validation in larger independent cohorts.

Among the individual body composition biomarkers evaluated, SMD was significantly associated with overall survival in the primary multivariable analysis. Each one-standard-deviation increase in SMD was associated with a reduction in mortality risk, whereas threshold-based definitions of myosteatosis were not significantly associated with survival outcomes in our cohort. These findings are consistent with previous studies suggesting that continuous assessment of muscle quality may provide greater prognostic information than dichotomous classifications based on predefined cutoffs [13,14,19,20,22,23]. While traditional radiologic definitions of sarcopenia have focused primarily on muscle quantity, increasing evidence indicates that muscle composition may represent a more biologically relevant marker of physiologic reserve and metabolic health [13,14,24].

Several mechanisms may explain the observed association between SMD and survival. Skeletal muscle is increasingly recognized as a central regulator of systemic metabolism and immune homeostasis. Progressive lipid infiltration of muscle tissue is associated with chronic inflammation, altered mitochondrial function, insulin resistance, anabolic resistance, and impaired secretion of myokines involved in immune regulation [19,20,23]. Consequently, reduced SMD may reflect a state of impaired immunometabolic fitness that limits the ability of patients to tolerate treatment and mount effective antitumor immune responses. In the setting of ICI therapy, where treatment efficacy depends on both tumor biology and preserved host immunity, these mechanisms may be particularly relevant.

In contrast to SMD, SMVI demonstrated limited prognostic value in our cohort. Although skeletal muscle depletion has historically been considered the cornerstone of radiologic sarcopenia assessment, the present findings suggest that muscle quantity alone may inadequately capture the biologic complexity of cancer-associated body composition alterations. Importantly, contemporary definitions of sarcopenia incorporate muscle strength in addition to measures of muscle quantity. As muscle strength was not available in our retrospective cohort, low SMVI should not be considered equivalent to clinically defined sarcopenia. Similar observations have been reported in studies suggesting that myosteatosis may be more strongly associated with clinical outcomes than conventional measures of skeletal muscle quantity in patients receiving ICIs [24,25,26]. Taken together, these findings support a shift from purely quantitative assessment of skeletal muscle toward more comprehensive evaluation of muscle composition and quality.

The prognostic impact of adiposity remains controversial in patients receiving ICIs. In the present study, higher VAVI was associated with a higher risk of death in the multivariable analysis, whereas SAVI was not significantly associated with OS. Neither VAVI nor SAVI was significantly associated with PFS. These findings may help reconcile some of the conflicting data surrounding the so-called obesity paradox. Several studies have reported improved outcomes among overweight or obese patients receiving immunotherapy, whereas others have demonstrated adverse effects associated with excess adiposity [15,16,27,28]. Such discrepancies may largely reflect the limitations of BMI, which does not distinguish between lean tissue and adipose tissue compartments and provides no information regarding fat distribution.

From a biological perspective, visceral adipose tissue is characterized by a pro-inflammatory secretory profile involving cytokines, adipokines, and other mediators capable of modulating immune function. Chronic visceral inflammation has been linked to T-cell dysfunction, altered macrophage polarization, and impaired antitumor immunity [15,16,21]. In the present study, higher VAVI was associated with a higher risk of death after adjustment for the prespecified clinical covariates, supporting the potential prognostic relevance of visceral fat distribution beyond conventional measures of adiposity. However, given the modest sample size and exploratory nature of the analysis, this association requires validation in independent cohorts.

One additional observation is that the exploratory integrated phenotype analysis showed numerically greater separation for OS than for PFS. However, the overall Kaplan–Meier comparisons were not statistically significant for either OS or PFS, and no consistent stepwise risk gradient was observed across the phenotype categories. This pattern may suggest that body composition characteristics are more closely related to overall prognosis than to initial disease control; however, this interpretation remains exploratory. Similar discrepancies between OS and PFS have been reported for other host-related biomarkers in patients treated with ICIs [7,25,27,29].

Beyond the biologic implications, the present study highlights the potential clinical utility of AI-based body composition analysis. CT-derived body composition assessment has emerged as a promising imaging biomarker in oncology, providing quantitative information on skeletal muscle and adipose tissue that may complement conventional clinical and molecular prognostic factors [30]. Historically, implementation of body composition biomarkers has been limited by the labor-intensive nature of manual segmentation techniques and concerns regarding reproducibility. Conventional approaches require expert annotation at predefined anatomic landmarks, most commonly L3, thereby limiting scalability in routine practice [9]. Recent advances in deep learning have enabled rapid, reproducible, and fully automated quantification of skeletal muscle and adipose tissue from routine CT examinations. Contemporary AI-based pipelines substantially reduce operator dependence while improving scalability and reproducibility, supporting the translation of body composition analysis into routine clinical workflows [17,18,31,32,33]. Moreover, emerging evidence indicates that these AI-based biomarkers remain reliable across different CT acquisition and reconstruction settings, further facilitating their integration into routine imaging workflows [32,34]. The clinical implications of these findings may be substantial. Current treatment decisions in aNSCLC are primarily guided by tumor-centered biomarkers such as PD-L1 expression and molecular profiling. However, these markers provide limited information regarding host fitness, metabolic reserve, or vulnerability to treatment-related complications. The incorporation of CT-derived body composition parameters into prognostic models may potentially contribute to the identification of patients at increased risk of poor outcomes despite receiving standard-of-care therapy. Integration of CT-derived body composition parameters with PD-L1 expression, circulating inflammatory markers, and molecular profiling may ultimately improve individualized risk stratification in advanced NSCLC.

Prospectively, automated CT-derived body composition measures could be embedded into clinical trials using routinely acquired baseline and longitudinal CT examinations, allowing standardized assessment without additional imaging burden. Such studies could evaluate whether SMD, VAVI, and related measures provide incremental prognostic information beyond established clinical and molecular factors and whether longitudinal changes are associated with treatment tolerance, treatment-related toxicity, functional decline, or survival. Importantly, AI-derived body composition metrics could also be investigated as stratification or enrichment variables in prospective trials of supportive care and prehabilitation strategies. For example, patients identified as having unfavorable body composition profiles could potentially be prospectively evaluated for targeted nutritional counseling, resistance and aerobic exercise programs, or combined multimodal prehabilitation interventions. Longitudinal assessment of these metrics may further help determine whether changes in skeletal muscle and adipose tissue during treatment are associated with response to such interventions or with subsequent clinical outcomes. However, whether AI-derived body composition measures can reliably identify patients who benefit from specific prehabilitation or nutritional interventions remains unknown and should be established in prospective interventional studies. Thus, the present findings should be considered hypothesis-generating rather than sufficient to support treatment selection or clinical intervention based on these metrics alone.

Several limitations should be acknowledged. First, the retrospective single-center design introduces the potential for selection bias and residual confounding. In addition, exclusion of patients without evaluable baseline CT imaging may have introduced further selection bias by restricting the study population to patients with imaging suitable for automated body composition analysis. Second, the relatively modest sample size limited statistical power for subgroup analyses and may have contributed to the lack of significance for some exploratory comparisons. Third, PFS analyses were limited by incomplete progression-related follow-up data and should therefore be interpreted cautiously. Fourth, the integrated phenotypes were derived using cohort-specific sex-stratified median values because validated volumetric thresholds for AI-derived SMVI and VAVI are currently unavailable. Furthermore, functional measures such as performance status, frailty, and muscle strength were not consistently available and therefore could not be incorporated into the analyses. Finally, external validation in independent cohorts is required before clinical implementation.

## 5. Conclusions

In this retrospective single-center cohort of patients with advanced NSCLC receiving first-line ICI-based therapy, CT-derived body composition measures were associated with overall survival. Higher skeletal muscle density was associated with lower mortality, whereas higher visceral adiposity was associated with a higher risk of death. The exploratory integrated phenotype analysis showed numerically favorable survival in the high-muscle/low-visceral-adiposity group; however, no consistent risk gradient across phenotype categories was observed. No individual CT-derived body composition parameter was significantly associated with progression-free survival. Given the modest sample size, cohort-specific sex-stratified median values used for phenotype classification, and retrospective design, these findings should be considered hypothesis-generating. External validation in larger prospective cohorts using standardized body composition thresholds is required before CT-derived body composition measures can be incorporated into clinical risk stratification.

## Figures and Tables

**Table 1:** **Baseline demographic and clinicopathological characteristics of the study population according to the integrated body composition phenotype.**

Characteristic	Total *N* = 103	High-Muscle/Low-Visceral-Adiposity *n* = 13	Intermediate Phenotype *n* = 77	Low-Muscle/High-Visceral-Adiposity *n* = 13	*p*-Value
**Age at treatment initiation, years**	65.0 (11.7)	62.2 (10.9)	64.6 (12.2)	70.1 (8.2)	0.148
**Sex**					**0.031**
Male	65 (63.1%)	11 (84.6%)	43 (55.8%)	11 (84.6%)	
Female	38 (36.9%)	2 (15.4%)	34 (44.2%)	2 (15.4%)	
**Smoking status**					**0.778**
Never	10 (9.7%)	2 (15.4%)	8 (10.4%)	0 (0.0%)	
Current	42 (40.8%)	5 (38.5%)	32 (41.6%)	5 (38.5%)	
Former	42 (40.8%)	6 (46.2%)	29 (37.7%)	7 (53.8%)	
Unknown	9 (8.7%)	0 (0.0%)	8 (10.4%)	1 (7.7%)	
**Histology**					**0.705**
Adenocarcinoma	64 (62.1%)	7 (53.8%)	48 (62.3%)	9 (69.2%)	
Squamous-cell carcinoma	27 (26.2%)	4 (30.8%)	19 (24.7%)	4 (30.8%)	
Other	12 (11.7%)	2 (15.4%)	10 (13.0%)	0 (0.0%)	
**PD-L1 tumor proportion score**					**0.397**
<1%	32 (32.7%)	5 (38.5%)	22 (30.1%)	5 (41.7%)	
1–49%	31 (31.6%)	4 (30.8%)	26 (35.6%)	1 (8.3%)	
≥50%	35 (35.7%)	4 (30.8%)	25 (34.2%)	6 (50.0%)	
**Stage**					**1.0**
IIIC	1 (1.0%)	0 (0.0%)	1 (1.3%)	0 (0.0%)	
IV	102 (99.0%)	13 (100.0%)	76 (98.7%)	13 (100.0%)	
**First-line treatment**					**1.0**
ICI monotherapy	22 (21.4%)	3 (23.1%)	16 (20.8%)	3 (23.1%)	
Chemoimmunotherapy	81 (78.6%)	10 (76.9%)	61 (79.2%)	10 (76.9%)	
**Metastatic burden, number of sites**	1.0 (1.0–2.0)	2.0 (1.8–2.0)	1.0 (1.0–2.0)	1.0 (1.0–2.0)	0.090

## Data Availability

The data presented in this study are available from the corresponding author upon reasonable request. The data are not publicly available due to privacy and ethical restrictions.
